# Potent bactericidal efficacy of copper oxide impregnated non-porous solid surfaces

**DOI:** 10.1186/1471-2180-14-57

**Published:** 2014-03-07

**Authors:** Alastair B Monk, Vikram Kanmukhla, Ken Trinder, Gadi Borkow

**Affiliations:** 1 Cupron Inc, 800 East Leigh Street, Suite 123, Richmond, VA 23219, USA; 2 EOS Surfaces, Norfolk, VA, USA

**Keywords:** Copper oxide, EPA registered, Antimicrobial surface, Environmental bioburden

## Abstract

**Background:**

The role of fomites and the environment in nosocomial infections is becoming widely recognized. In this paper we discuss the use of Cupron copper oxide impregnated non-porous solid surface in the hospital setting and present *in vitro* testing data via USA Environmental Protection Agency (EPA) approved testing protocols that demonstrate the efficacy of these products to assist in reduction in environmental contamination and potentially nosocomial infections.

**Results:**

The two countertops tested passed all the acceptance criteria by the EPA (>99.9% kill within 2 hours of exposure) killing a range of bacterial pathogens on the surface of the countertops even after repeated exposure of the countertops to the pathogen, and multiple wet and dry abrasion cycles.

**Conclusions:**

Cupron enhanced EOS countertops thus may be an important adjunct to be used in hospital settings to reduce environmental bioburden and potentially nosocomial infections.

## Background

Hospital Acquired Infections (HAI) have exacted a heavy toll worldwide with over 2 million patients annually contracting an infection in the US [1], being one of the leading causes of death in the US behind cancer and strokes [2]. In Europe, out of 3 million HAI [3] approximately 50,000 resulted in death [4], and in Australia more than 177,000 HAI occur per year [5] whilst in the province of Quebec, Canada the rate of HAI are estimated to be around 11% [6]. The HAI rates in developing countries are significantly higher [7-9]. According to the USA Center for Disease Control (CDC) some of the predominant HAI organisms are *Staphylococcus aureus, Pseudomonas aeruginosa*, and *Enterobacter* species [10]. Methicillin resistant *S. aureus* accounts for 50% of HAI associated with multidrug resistant pathogens [10]. The Extended Prevalence of Infection in Intensive Care (EPIC II) study demonstrated a 50% HAI rate in ICU patients sampled from over 75 countries and two of the most predominant organisms were resistant Staphylococci and *P. aeruginosa*[11]. HAI are associated with considerable mortality, morbidity and costs [2,12]. Recent intervention efforts including improvement of national surveillance, use of aggressive antibiotic control programs, healthcare staff education for improved hygiene, isolation of infected patients, use of disposable equipment, cleaning and disinfection of environmental surfaces and equipment, improvement of cleaning equipment and sanitary facilities, increase in nursing and janitorial resources and better nutrition [13-17], have been shown to reduce HAI rates. However further supplemental interventions are required. The link between contaminated hard surfaces to HAI has been demonstrated [18-28] and an antimicrobial protected touch surface would assist in reduction of pathogen buildup upon touch surfaces as long as that activity can be indisputably demonstrated.

Thus, our objective was to demonstrate the efficacy of two solid surface countertops incorporating copper (I) oxide in killing a range of pathogens according to protocols that were approved by the USA Environmental Protection Agency (EPA).

## Methods

### Bacterial strains

The following bacterial strains were tested: *Staphylococcus aureus* (ATCC 6538); *Enterobacter aerogenes* (ATCC 13048); *Pseudomonas aeruginosa* (ATCC 15442); Methicillin resistant *Staphylococcus aureus* (MRSA)(ATCC 33592); and *Escherichia coli* 0157:H7 (ATCC 35150).

### Materials

**Figure 1:**
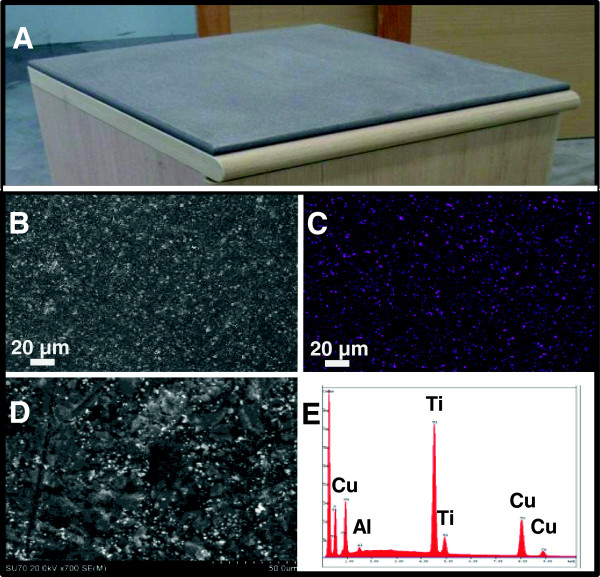
**SEM pictures and EDS analysis of a representative countertop containing copper oxide particles. A**. A representative picture of a tested countertop impregnated with copper oxide; **B**. A SEM imaging of the Countertop (white dots indicating copper oxide particles; **C**. EDS imaging of the Countertop (purple dots indicating the copper oxide); **D**. cut through SEM imaging of the Countertop (white dots indicating copper oxide particles); and **E**. corresponding EDS spectra of D, showing a peak corresponding to copper.

The studied countertops were composed of homogenous blends of polyester, acrylic alloys and fillers, inert pigment and dyes, with (test samples) or without (control samples) Cupron’s 16% copper (I) oxide weight/weight. Three and two separate manufacturing lots of the test and control countertop samples were tested, respectively. A total of 1500 pieces, cut into one inch by one inch squares (Figure 1), 300 per each manufacturing lot, were tested. The countertops were examined by Scanning Electron Microscopy (SEM) and Energy-dispersive X-ray spectroscopy (EDS) by using Hitachi FE-SEM SU-70. The Cupron Enhanced EOS Surface is a novel polymeric solid surface that has all the properties of a solid surface including hardness, firmness, and the ability to be easily cleaned and shaped or fashioned with the antimicrobial ability of copper. The surface can be easily refinished and repaired in the event of damage or aesthetic appeal. The surfaces are currently available in two color choices due to the addition of pigments to alter the color of the surfaces at the time of manufacture. The surface is produced by mixing a blend of acrylic and polyester resins with copper oxide and pigments, which is then heated until liquified and poured into casting molds. The material is allowed to cure allowing the polymerization of the material to produce a solid surface which can then be cut and shaped to produce a final product or installed surface.

### Biocidal testing protocols

The biocidal testing of the countertops was conducted by an independent laboratory, MicroBiotest, a division of Microbac Laboratories, Inc. Sterling, VA, using Good Laboratory Practice (GLP) according to protocols pre-approved by the USA EPA.

#### Protocol 1- sanitizer activity

The carriers were cleaned with 70% isopropyl alcohol, rinsed with deionized water, and allowed to air dry. After steam sterilization for 15 minutes at 121°C, each carrier was placed into a plastic Petri dish matted with two pieces of filter paper using sterile forceps. Two or three lots of manufacturing of each test material were tested per microorganism. Five replicates of each material were used in each test. Organisms from stock cultures were transferred to Tryptic Soy Broth and incubated for 24 hours at 35-37°C (25-30°C for ATCC 13048). Two loopfuls of culture were transferred consecutively daily for three days for the inoculation stocks and the pellicle of bacteria were aspirated. Daily transfers were done for at least 3 consecutive days but for no more than 10 days. To this culture 0.25 ml of heat-inactivated fetal bovine serum (FBS) and 0.05 ml of Triton X-100 were added to 4.7 ml bacteria suspension to yield 5% FBS and 0.01% Triton X-100 organic soil load. The challenge microorganism titer was determined by serially diluting a final 48 hour culture using phosphate buffered solution (PBS) and selected dilutions were plated in duplicate using Tryptic Soy Agar (TSA) pour plates. Carriers were inoculated with 0.02 ml of the 48 hour culture. The bacterial inoculum per experiment is detailed in Table 1. All control plates were incubated in parallel to the test plates. The inoculum was spread to within ~1/8 inch of the control or test carrier before air drying for 20–40 minutes at 35-37°C and 38-42% relative humidity. After 120 minutes exposure at 21°C, the carriers were transferred to 20 ml neutralizer solution (2x Letheen broth [29]) and sonicated for 5 minutes and rotated to mix. Within one hour serial dilutions (10^−1^ to 10^−4^) were made in PBS and plated using TSA and incubated for 48 hours at 35-37°C for colony observation and enumeration, taking into account also the 20 fold dilution used to retrieve the bacteria from the carriers. The following controls were performed: culture purity control - each prepared culture was streaked using TSA for purity control; organic soil purity control - duplicate 1 ml aliquots of organic soil were plated in TSA pour plates for sterility control; neutralizer sterility control – a jar containing the neutralizer was incubated with the test plates and observed for growth or no growth; carrier sterility control – an uninoculated test (per lot) and control carrier was put in independent jars containing the neutralizer, incubated and observed for growth or no growth; carrier viability control – for each challenge microorganism, a single inoculated control carrier was subcultured in a jar containing the neutralizer, incubated and the neutralizer observed for growth or no growth; and neutralization confirmation control – for each challenge microorganisms, per lot of the test article, a single sterile test carrier was put in individuals jars containing 20 ml of the neutralizer. To each jar a 1 ml aliquot of the diluted inoculum was added to reach ~100 colony forming units (CFU)/ml in the neutralizer. The jar was mixed and the 1 ml inoculum was removed and plated in duplicate.

**Table 1:** CFU recovered from control samples

**Microorganism**	**Protocol**	**Replicate**	**Inoculum**	**Titers recovered**	**Average**
			**CFU/carrier**	**CFU/carrier**	**CFU/carrier**
*S. aureus*	1	1	0.22 × 10^8^	8.4 × 10^5^	7.5 × 10^5^
		2		7.0 × 10^5^	
		3		7.2 × 10^5^	
	2 - initial	1	0.42 × 10^8^	1.1 × 10^6^	1.3 × 10^6^
		2		1.6 × 10^6^	
		3		1.2 × 10^6^	
		4		1.3 × 10^6^	
	2 - Final	1	0.46 × 10^8^	9.9 × 10^5^	1.1 × 10^6^
		2		1.1 × 10^6^	
		3		1.3 × 10^6^	
		4		1.0 × 10^6^	
	3 - 2 hours	1	0.38 × 10^8^	8.2 × 10^5^	9.3 × 10^5^
		2		1.0 × 10^6^	
		3		9.4 × 10^5^	
	3 - 6 hours	1		2.0 × 10^6^	1.8 × 10^6^
		2		1.8 × 10^6^	
		3		1.7 × 10^6^	
	3 - 12 hours	1		2.5 × 10^6^	2.5 × 10^6^
		2		2.4 × 10^6^	
		3		3.7 × 10^6^	
	3 - 18 hours	1		3.7 × 10^6^	3.6 × 10^6^
		2		3.6 × 10^6^	
		3		3.5 × 10^6^	
	3 - 24 hours	1		4.6 × 10^6^	4.6 × 10^6^
		2		4.6 × 10^6^	
		3		4.5 × 10^6^	
*E. aerogenes*	1	1	0.38 × 10^9^	7.4 × 10^6^	7.9 x10^6^
		2		8.8 × 10^6^	
		3		7.6 × 10^6^	
	2 - initial	1	0.22 × 10^9^	1.1 × 10^6^	1.1 × 10^6^
		2		1.0 × 10^6^	
		3		1.2 × 10^6^	
		4		1.1 × 10^6^	
	2 - Final	1	0.4 × 10^9^	1.5 × 10^6^	1.2 × 10^6^
		2		1.4 × 10^6^	
		3		8.3 × 10^5^	
		4		1.1 × 10^6^	
	3 - 2 hours	1	0.38 × 10^9^	2.0 × 10^6^	2.0 × 10^6^
		2		2.1 × 10^6^	
		3		2.0 × 10^6^	
	3 - 6 hours	1		3.8 × 10^6^	3.9 × 10^6^
		2		3.9 × 10^6^	
		3		3.9 × 10^6^	
	3 - 12 hours	1		5.1 × 10^6^	4.7 × 10^6^
		2		5.4 × 10^6^	
		3		3.6 × 10^6^	
	3 - 18 hours	1		4.8 × 10^6^	5.6 × 10^6^
		2		6.8 × 10^6^	
		3		5.2 × 10^6^	
	3 - 24 hours	1		8.5 × 10^6^	7.9 × 10^6^
		2		8.4 × 10^6^	
		3		6.8 × 10^6^	
MRSA	1	1	0.38 × 10^9^	7.4 × 10^5^	8.5 × 10^5^
		2		9.8 × 10^5^	
		3		8.2 × 10^5^	
	2 - initial	1	0.36 × 10^8^	7.3 × 10^5^	7.5 × 10^5^
		2		9.5 × 10^5^	
		3		6.6 × 10^5^	
		4		6.5 × 10^5^	
	2 - Final	1	0.32 × 10^8^	5.6 × 10^5^	6.9 × 10^5^
		2		5.7 × 10^5^	
		3		8.0 × 10^5^	
		4		8.2 × 10^5^	
	3 - 2 hours	1	0.26 × 10^8^	4.0 × 10^5^	4.0 × 10^5^
		2		3.8 × 10^5^	
		3		4.2 × 10^5^	
	3 - 6 hours	1		8.6 × 10^5^	8.8 × 10^5^
		2		9.8 × 10^5^	
		3		7.9 × 10^5^	
	3 - 12 hours	1		9.9 × 10^5^	1.0 × 10^6^
		2		1.2 × 10^6^	
		3		9.1 × 10^5^	
	3 - 18 hours	1		1.8 × 10^6^	1.7 × 10^6^
		2		1.6 × 10^6^	
		3		1.7 × 10^6^	
	3 - 24 hours	1		1.8 × 10^6^	1.8 × 10^6^
		2		1.8 × 10^6^	
		3		1.7 × 10^6^	
*P. aeruginosa*	1	1	0.2 × 10^8^	6.8 × 10^6^	7.0 × 10^6^
		2		7.4 × 10^6^	
		3		6.9 × 10^6^	
	2 - initial	1	0.2 × 10^9^	1.0 × 10^6^	1.3 × 10^6^
		2		1.4 × 10^6^	
		3		1.4 × 10^6^	
		4		1.5 × 10^6^	
	2 - Final	1	0.34 × 10^9^	2.4 × 10^6^	2.0 × 10^6^
		2		1.9 × 10^6^	
		3		1.6 × 10^6^	
		4		2.0 × 10^6^	
	3 - 2 hours	1	0.3 × 10^9^	2.6 × 10^5^	2.5 × 10^5^
		2		2.5 × 10^5^	
		3		2.5 × 10^5^	
	3 - 6 hours	1		5.2 × 10^5^	5.2 × 10^5^
		2		5.3 × 10^5^	
		3		5.3 × 10^5^	
	3 - 12 hours	1		7.2 × 10^5^	7.2 × 10^5^
		2		7.1 × 10^5^	
		3		7.4 × 10^5^	
	3 - 18 hours	1		9.8 × 10^5^	9.6 × 10^5^
		2		9.5 × 10^5^	
		3		9.6 × 10^5^	
	3 - 24 hours	1		9.8 × 10^5^	9.7 × 10^5^
		2		9.2 × 10^5^	
		3		1.0 × 10^6^	
*E. coli* O157:H7	1	1	0.36 × 10^9^	6.4 × 10^6^	6.6 × 10^6^
		2		6.7 × 10^6^	
		3		6.6 × 10^6^	
	2 - initial	1	0.24 × 10^9^	1.5 × 10^6^	1.1 × 10^6^
		2		8.3 × 10^6^	
		3		1.1 × 10^6^	
		4		1.1 × 10^6^	
	2 - Final	1	0.22 × 10^9^	7.8 × 10^5^	9.4 × 10^5^
		2		8.0 × 10^5^	
		3		1.2 × 10^6^	
		4		9.9 × 10^5^	
	3 - 2 hours	1	0.36 × 10^9^	2.5 × 10^5^	2.6 × 10^5^
		2		2.6 × 10^5^	
		3		2.7 × 10^5^	
	3 - 6 hours	1		5.2 × 10^5^	5.3 × 10^5^
		2		5.2 × 10^5^	
		3		5.4 × 10^5^	
	3 - 12 hours	1		7.9 × 10^5^	7.7 × 10^5^
		2		7.7 × 10^5^	
		3		7.6 × 10^5^	
	3 - 18 hours	1		1.0 × 10^6^	1.0 × 10^6^
		2		1.1 × 10^6^	
		3		1.0 × 10^6^	
	3 - 24 hours	1		1.2 × 10^6^	1.2 × 10^6^
		2		1.2 × 10^6^	
		3		1.2 × 10^6^	

#### Protocol 2- residual sanitizer activity

A sanitization test was followed as described above (Protocol 1) using 4 replicates per material. Post this initial test a Gardner apparatus was used to simulate surface wear of the test and control samples. The abrasion tester was used at a speed of 2.25 to 2.5 for a total contact time of 4–5 seconds for one complete cycle. A wear cycle equals one pass to the left and a return pass to the right. After a minimum of 15 minutes after the wear cycle each carrier was reinoculated as described above and dried for a minimum of 30 minutes. After each set of surface wear, absolute ethanol was used to sterilize the apparatus and the foam liner and cotton cloth were changed after each wear test. Wet cycles and dry cycles were alternated and for wet wear cycles the boat assembly included a new foam liner and dry cotton cloth sprayed with sterile deionized water using a preval sprayer from a distance of 75±1 cm for not more than one second. At least 24 hours passed between the initial inoculation and final sanitizer. Overall 12 wear cycles were completed before sanitizer activity was assessed using the method outlined above. All the controls as outlined for Protocol 1 were performed.

#### Protocol 3- continuous bacterial reduction

A sanitization test was followed as described above (Protocol 1) using 5 replicates per each material tested. The carriers were consecutively inoculated for 8 times by adding the challenge microorganism at 0, 3, 6, 9, 12, 15, 18 and 21 hours. Efficacy was assessed at 2, 6, 12, 18 and 24 hours, which corresponds to 1, 2, 4, 6, and 8 inoculations. After exposure the carriers were transferred to a neutralizer solution and sonicated and rotated to mix. Within one hour, serial dilutions (10^−1^ to 10^−4^) were spread on plates using appropriate media and incubated for 48 hours for colony observation and enumeration. All the controls as outlined for Protocol 1 were performed.

## Results

The challenge microorganisms were confirmed for purity by Gram stain and colony morphology. Controls demonstrated that the organic soil, carrier and neutralizing medium were sterile. The neutralizing solution itself did not show any bacterial inhibition. The bacterial titers (actual CFU after taking into consideration the relevant dilutions) recovered from the control samples following the different protocols, which included air drying, sonication, and recovering the bacteria from the exposed carrier, are summarized in Table 1. Tables 2, 3 and 4 show the bacterial titers recovered from the Test samples following the various protocols and the percent reduction in the bacterial titers recovered as compared to the mean bacterial titers recovered from the control samples. All experiments conducted with the copper oxide impregnated countertops demonstrated over a 3 log (>99.9%) reduction against all organisms tested, as compared to the control countertops without copper (Tables 2, 3 and 4). Out of the 192 data points obtained (average of 4 or 5 replicates each) for the test countertops, there were only two exceptions for the continuous sanitizer activity test - with a 99.8% and 99.2% reductions against *Pseudomonas aeruginosa* (Table 4), which exceeds the 99% reduction requirement set up by the EPA for continuous efficacy kill rates*.* As determined by SEM and EDS analysis, copper oxide particles are homogenously distributed within (Figure 1D and E) and throughout the surface (Figure 1B and C) of the test countertops.

**Table 2:** Results from Protocol 1- Sanitizer Activity

**Countertop**	**Organism**	CFU/ Recovered from control samples*	**Lot**	**CFU recovered from test samples**	% reduction^**^
Test 1	*S. aureus*	7.5 × 10^5^	1	<1;<1;5;<1;<1	>99.9
			2	18;<1;11;<1;22	>99.9
			3	<1;<1;<1;<1;<1	>99.9
	*E. aerogenes*	7.9 × 10^6^	1	1200;790;50;1200;<1	>99.9
			2	760;840;1200;800;620	>99.9
			3	<1;620;<1;500;<1	>99.9
	*MRSA*	8.5 × 10^5^	1	<1;<1;<1;<1;<1	>99.9
			2	<1;<1;<1;<1;<1	>99.9
	*P. aeruginosa*	7 × 10^6^	1	90;580;<1;50;160	>99.9
			2	440;<1;400;<1;<1	>99.9
	*E. coli 0157:H7*	6.6 × 10^6^	1	470;690;450;480;380	>99.9
			2	560;360;320;390;360	>99.9
Test 2	*S. aureus*	7.5 × 10^5^	1	<1;50;<1;80;<1	>99.9
			2	280;<1;70;<1;<1	>99.9
			3	<1;<1;<1;<1;<1	>99.9
	*E. aerogenes*	7.9 × 10^6^	1	70;540;140;650;120	>99.9
			2	240;750;240;460;410	>99.9
			3	770;610;410;230;450	>99.9
	*MRSA*	8.5 × 10^5^	1	<1;<1;<1;<1;<1	>99.9
			2	<1;<1;<1;<1;<1	>99.9
	*P. aeruginosa*	7.0 × 10^6^	1	820;740;600;880;890	>99.9
			2	930;840;730;870;990	>99.9
	*E. coli 0157:H7*	6.6 × 10^6^	1	640;720;300;700;700	>99.9
			2	660;540;490;760;300	>99.9

*Values taken from Table 1.

**Compared to control, each number represents an average of 5 replicates per manufacturing lot. Either 2 or 3 lots were examined per organism.

**Table 3:** Results from protocol 2- residual sanitizer efficacy

**Countertop**	**Organism**	CFU recovered from control samples*	**Lot**	**CFU recovered from test samples**	% reduction**
Test 1- Initial	*S. aureus*	1.3 × 10^6^	1	<1.5;<1.5;<1.5;<1.5	>99.9
			2	<1.5;<1.5;<1.5;<1.5	>99.9
			3	<1.5;<1.5;<1.5;<1.5	>99.9
	*E. aerogenes*	1.1 × 10^6^	1	<1.5;<1.5;<1.5;<1.5	>99.9
			2	<1.5;<1.5;<1.5;<1.5	>99.9
			3	390;<1.5;<1.5;<1.5	>99.9
	*MRSA*	7.5 × 10^5^	1	<1.5;<1.5;<1.5;<1.5	>99.9
			2	<1.5;<1.5;<1.5;<1.5	>99.9
	*P. aeruginosa*	1.3 × 10^6^	1	<1.5;<1.5;<1.5;<1.5	>99.9
			2	<1.5;<1.5;<1.5;90	>99.9
	*E. coli 0157:H7*	1.1 × 10^6^	1	<1.5;<1.5;<1.5;<1.5	>99.9
			2	<1.5;<1.5;<1.5;<1.5	>99.9
Test 1- Final	*S. aureus*	1.1 × 10^6^	1	<1.5;<1.5;<1.5;<1.5	>99.9
			2	<1.5;<1.5;<1.5;<1.5	>99.9
			3	<1.5;<1.5;<1.5;<1.5	>99.9
	*E. aerogenes*	1.2 × 10^6^	1	<1.5;<1.5;<1.5;<1.5	>99.9
			2	<1.5;<1.5;<1.5;<1.5	>99.9
			3	<1.5;<1.5;<1.5;<1.5	>99.9
	*MRSA*	6.9 × 10^5^	1	<1.5;<1.5;<1.5;<1.5	>99.9
			2	<1.5;<1.5;<1.5;<1.5	>99.9
	*P. aeruginosa*	2.0x10^6^	1	<1.5;<1.5;<1.5;<1.5	>99.9
			2	<1.5;<1.5;<1.5;<1.5	>99.9
	*E. coli 0157:H7*	9.4 × 10^5^	1	<1.5;<1.5;<1.5;<1.5	>99.9
			2	<1.5;<1.5;<1.5;<1.5	>99.9
Test 2- Initial	*S. aureus*	1.3 × 10^6^	1	4.5;<1.5;<1.5;<1.5	>99.9
			2	<1.5;<1.5;<1.5;200	>99.9
			3	<1.5;<1.5;<1.5;240	>99.9
	*E. aerogenes*	1.1 × 10^6^	1	<1.5;60;180;<1.5	>99.9
			2	9;150;420;<1.5	>99.9
			3	<1.5;<1.5;<1.5;<1.5	>99.9
	*MRSA*	7.6 × 10^5^	1	<1.5;<1.5;<1.5;<1.5	>99.9
			2	<1.5;<1.5;<1.5;<1.5	>99.9
	*P. aeruginosa*	1.3 × 10^6^	1	150;<1.5;9;230	>99.9
			2	450;570;<1.5;<1.5	>99.9
	*E. coli 0157:H7*	1.1 × 10^6^	1	<1.5;60;180;<1.5	>99.9
			2	90;150;420;<1.5	>99.9
Test 2- Final	*S. aureus*	1.1 × 10^6^	1	<1.5;<1.5;<1.5;<1.5	>99.9
			2	<1.5;330;<1.5;<1.5	>99.9
			3	<1.5;<1.5;<1.5;<1.5	>99.9
	*E. aerogenes*	1.2 × 10^6^	1	380;<1.5;<1.5;<1.5	>99.9
			2	<1.5;<1.5;<1.5;320	>99.9
			3	<1.5;<1.5;<1.5;<1.5	>99.9
	*MRSA*	6.9 × 10^5^	1	<1.5;<1.5;<1.5;<1.5	>99.9
			2	<1.5;<1.5;<1.5;<1.5	>99.9
	*P. aeruginosa*	2.0 × 10^6^	1	<1.5;<1.5;<1.5;<1.5	>99.9
			2	<1.5;<1.5;<1.5;<1.5	>99.9
	*E. coli 0157:H7*	9.4 × 10^5^	1	<1.5;<1.5;<1.5;<1.5	>99.9
			2	<1.5;<1.5;<1.5;<1.5	>99.9

*Values taken from Table 1.

**Compared to control, each number represents an average of 4 replicates per manufacturing lot. Either 2 or 3 lots were examined per organism.

**Table 4:** Results from protocol 3- continuous self sanitizing activity

**Countertop**	**Organism**	**CFU recovered from control samples**	**Lot**	**CFU recovered from test samples**	% reduction**
Test 1–2 hours	*S. aureus*	9.3 × 10^5^	1	220;340;500;670;290	>99.9
			2	420;270;290;320;220	>99.9
			3	380;420;340;290;270	>99.9
	*E. aerogenes*	2.0 × 10^6^	1	11;220;<1<1<1	>99.9
			2	<1;100;220;<1;<1	>99.9
			3	80;40;170;80	>99.9
	*MRSA*	4.0 × 10^5^	1	<1;<1;<1;<1;<1	>99.9
			2	<1;<1;<1;<1;<1	>99.9
	*P. aeruginosa*	2.5 × 10^5^	1	480;370;480;180;120	99.9
			2	420;480;240;450;360	99.8
	*E. coli 0157:H7*	2.6 × 10^5^	1	<1;<1;<1;<1;<1	>99.9
			2	140;<1;<1;<1;150	99.9
Test 1–6 hours	*S. aureus*	1.8 × 10^6^	1	<1;<1;<1;<1;<1	>99.9
			2	<1;<1;<1;<1;<1	>99.9
			3	<1;<1;<1;<1;<1	>99.9
	*E. aerogenes*	3.9 × 10^6^	1	<1;<1;<1;<1;<1	>99.9
			2	<1;<1;<1;<1;<1	>99.9
			3	<1;<1;<1;<1;<1	>99.9
	*MRSA*	8.8 × 10^5^	1	<1;<1;<1;<1;<1	>99.9
			2	<1;<1;<1;<1;<1	>99.9
	*P. aeruginosa*	5.2 × 10^5^	1	<1;<1;<1;<1;<1	>99.9
			2	<1;170;<1;<1;<1	>99.9
	*E. coli 0157:H7*	5.3 × 10^5^	1	<1;<1;<1;<1;<1	>99.9
			2	<1;<1;<1;<1;<1	>99.9
Test 1–12 hours	*S. aureus*	2.5 × 10^6^	1	<1;<1;<1;<1;<1	>99.9
			2	<1;<1;<1;<1;<1	>99.9
			3	<1;<1;<1;<1;<1	>99.9
	*E. aerogenes*	4.7 × 10^6^	1	<1;<1;<1;<1;<1	>99.9
			2	<1;<1;<1;<1;<1	>99.9
			3	<1;<1;<1;<1;<1	>99.9
	*MRSA*	1.0 × 10^6^	1	<1;<1;<1;<1;<1	>99.9
			2	<1;<1;<1;<1;<1	>99.9
	*P. aeruginosa*	7.2 × 10^5^	1	<1;<1;<1;<1;<1	>99.9
			2	<1;<1;<1;<1;<1	>99.9
	*E. coli 0157:H7*	7.7 × 10^5^	1	<1;<1;<1;<1;<1	>99.9
			2	<1;<1;<1;<1;<1	>99.9
Test 1–18 hours	*S. aureus*	3.6 × 10^6^	1	<1;<1;<1;<1;<1	>99.9
			2	<1;<1;<1;<1;<1	>99.9
			3	<1;<1;<1;<1;<1	>99.9
	*E. aerogenes*	5.6 × 10^6^	1	<1;<1;<1;<1;<1	>99.9
			2	<1;<1;<1;<1;<1	>99.9
			3	<1;<1;<1;<1;<1	>99.9
	*MRSA*	1.7 × 10^6^	1	<1;<1;<1;<1;<1	>99.9
			2	<1;<1;<1;<1;<1	>99.9
	*P. aeruginosa*	9.6 × 10^5^	1	<1;<1;<1;<1;<1	>99.9
			2	<1;<1;<1;<1;<1	>99.9
	*E. coli 0157:H7*	1.0 × 10^6^	1	<1;<1;<1;<1;<1	>99.9
			2	<1;<1;<1;<1;<1	>99.9
Test 1–24 hours	*S. aureus*	4.6 × 10^6^	1	<1;<1;<1;<1;<1	>99.9
			2	<1;<1;<1;<1;<1	>99.9
			3	<1;<1;<1;<1;<1	>99.9
	*E. aerogenes*	7.9 × 10^6^	1	<1;<1;<1;<1;<1	>99.9
			2	<1;<1;<1;<1;<1	>99.9
			3	<1;<1;<1;<1;<1	>99.9
	*MRSA*	1.8 × 10^6^	1	<1;<1;<1;<1;<1	>99.9
			2	<1;<1;<1;<1;<1	>99.9
	*P. aeruginosa*	9.7 × 10^5^	1	<1;<1;<1;<1;<1	>99.9
			2	<1;<1;<1;<1;<1	>99.9
	*E. coli 0157:H7*	1.2 × 10^6^	1	<1;<1;<1;<1;<1	>99.9
			2	<1;<1;<1;<1;<1	>99.9
Test 2–2 hours	*S. aureus*	9.3 × 10^5^	1	760;580;770;730;550	>99.9
			2	780;770;520;540;460	>99.9
			3	480;420;420;450;410	>99.9
	*E. aerogenes*	2.0 × 10^6^	1	250;240;460;250;280	>99.9
			2	620;640;330;340;260	>99.9
			3	360;240;280;220;270	>99.9
	*MRSA*	4.0 × 10^5^	1	<1;<1;<1;<1;<1	>99.9
			2	<1;<1;<1;<1;<1	>99.9
	*P. aeruginosa*	2.5 × 10^5^	1	260;200;540;200;400	99.9
			2	200;410;560;280;680	99.2
	*E. coli 0157:H7*	2.6 × 10^5^	1	<1;130;210;<1;30	>99.9
			2	440;250;170;390;130	>99.9
Test 2–6 hours	*S. aureus*	1.8 × 10^6^	1	280;260;330;230;700	>99.9
			2	320;300;220;260;200	>99.9
			3	160;120;100;140;180	>99.9
	*E. aerogenes*	3.9 × 10^6^	1	<1;<1;<1;<1;<1	>99.9
			2	<1;<1;<1;<1;<1	>99.9
			3	<1;<1;<1;<1;<1	>99.9
	*MRSA*	8.8 × 10^5^	1	<1;<1;<1;<1;<1	>99.9
			2	<1;<1;<1;<1;<1	>99.9
	*P. aeruginosa*	5.2 × 10^5^	1	<1;<1;<1;<1;<1	>99.9
			2	<1;<1;<1;<1;<1	>99.9
	*E. coli 0157:H7*	5.3 × 10^5^	1	<1;<1;<1;<1;<1	>99.9
			2	<1;<1;<1;<1;<1	>99.9
Test 2–12 hours	*S. aureus*	2.5 × 10^6^	1	<1;<1;<1;<1;<1	>99.9
			2	<1;<1;<1;<1;<1	>99.9
			3	<1;<1;<1;<1;<1	>99.9
	*E. aerogenes*	4.7 × 10^6^	1	<1;<1;<1;<1;<1	>99.9
			2	<1;<1;<1;<1;<1	>99.9
			3	<1;<1;<1;<1;<1	>99.9
	*MRSA*	1.0 × 10^6^	1	<1;<1;<1;<1;<1	>99.9
			2	<1;<1;<1;<1;<1	>99.9
	*P. aeruginosa*	7.2 × 10^5^	1	<1;<1;<1;<1;<1	>99.9
			2	<1;<1;<1;<1;<1	>99.9
	*E. coli 0157:H7*	7.7 × 10^5^	1	<1;<1;<1;<1;<1	>99.9
			2	<1;<1;<1;<1;<1	>99.9
Test 2–18 hours	*S. aureus*	3.6 × 10^6^	1	<1;<1;<1;<1;<1	>99.9
			2	<1;<1;<1;<1;<1	>99.9
			3	<1;<1;<1;<1;<1	>99.9
	*E. aerogenes*	5.6 × 10^6^	1	<1;<1;<1;<1;<1	>99.9
			2	<1;<1;<1;<1;<1	>99.9
			3	<1;<1;<1;<1;<1	>99.9
	*MRSA*	1.7 × 10^6^	1	<1;<1;<1;<1;<1	>99.9
			2	<1;<1;<1;<1;<1	>99.9
	*P. aeruginosa*	9.6 × 10^5^	1	<1;<1;<1;<1;<1	>99.9
			2	<1;<1;<1;<1;<1	>99.9
	*E. coli 0157:H7*	1.0 × 10^6^	1	<1;<1;<1;<1;<1	>99.9
			2	<1;<1;<1;<1;<1	>99.9
Test 2–24 hours	*S. aureus*	4.6 × 10^6^	1	<1;<1;<1;<1;<1	>99.9
			2	<1;<1;<1;<1;<1	>99.9
			3	<1;<1;<1;<1;<1	>99.9
	*E. aerogenes*	7.9 × 10^6^	1	<1;<1;<1;<1;<1	>99.9
			2	<1;<1;<1;<1;<1	>99.9
			3	<1;<1;<1;<1;<1	>99.9
	*MRSA*	1.8 × 10^6^	1	<1;<1;<1;<1;<1	>99.9
			2	<1;<1;<1;<1;<1	>99.9
	*P. aeruginosa*	9.7 × 10^5^	1	<1;<1;<1;<1;<1	>99.9
			2	<1;<1;<1;<1;<1	>99.9
	*E. coli 0157:H7*	1.2 × 10^6^	1	<1;<1;<1;<1;<1	>99.9
			2	<1;<1;<1;<1;<1	>99.9

*Values taken from Table 1.

**Compared to control, each number represents an average of 5 replicates per manufacturing lot. Either 2 or 3 lots were examined per organism.

## Discussion

Bacteria can persist on inanimate surfaces for months [30] and can be a potential source for outbreaks of nosocomial infections [18,19,27]. Thus using self-sanitizing surfaces can be a very important adjunct in the fight against nosocomial pathogens [31]. The data collected under GLP independent testing using a predefined concentration of cultivated ATCC referenced bacterial strains, demonstrated the antimicrobial properties of Cupron copper oxide impregnated countertops. Protocol number 1 tested the capacity of copper oxide infused countertops to kill a number of cultivated pathogens (Table 2) under conditions prescribed by the US EPA for the in vitro testing of the antimicrobial efficacy of copper oxide particles suspended in a plastic matrix. The organisms tested constitute a broad representation of current HAI organisms, and with over a three log reduction (>99.9%) achieved within 2 hours of exposure the authors conclude that these copper oxide infused countertops can be an additional tool for bioburden reduction and potentially reducing the risk of HAI. Importantly, as demonstrated by using Protocol 2, simulating prolonged surface wear, the countertops continue to be highly efficacious even after 12 consecutive wet and dry wear and inoculation cycles (Table 3), simulating surface abrasion that occurs due to cleaning and use. Despite the erosion of the countertops’ surface, there was no reduction in biocidal efficacy. This is explained by the distribution of the copper oxide particles throughout the matrix, on and within the surface (Figure 1), and the appearance of “new” particles on the surface as the countertop surface is eroded. This property of the countertops practically endows them with biocidal properties for the life of the product. Protocol 3 demonstrated that the countertops are efficacious to consecutive bacterial inoculations (Table 4) in the same exact spot, indicating that the countertops do not lose their biocidal efficacy following bacterial kill, but maintain this biocidal property continuously.

Copper has a long history as an antimicrobial and preventative measure and metallic copper countertops have previously been approved for EPA public health claims [32]. Field trials of these countertops have demonstrated the reduction in bioburden in a variety of clinical settings [33-37] and a reduction in the risk of infections [38,39].

Based on the data presented in this publication, Cupron Enhanced EOS Surfaces infused with copper have been approved for public health claims relating to their anti bacterial efficacy. Some of the approved health claims are a) “This surface continuously reduces bacterial* contamination achieving a 99.9% reduction within two hours of exposure.”; b) “This surface kills greater than 99.9% of Gram negative and Gram positive bacteria* within two hours of exposure.”; c) “This surface kills greater than 99.9% of bacteria* within two hours and continues to kill 99% of bacteria* even after repeated contamination.”; and d) “This surface helps inhibit the buildup and growth of bacteria* within two hours of exposure between routine cleaning and sanitizing steps”. *Testing demonstrates effective antibacterial activity against *Staphylococcus aureus* (ATCC 6538), *Enterobacter aerogenes* (ATCC 13048), MRSA (ATCC 33592), *Escherichia coli* O157:H7 (ATCC 35150) and *Pseudomonas aeruginosa* (ATCC 15442).

These non-porous Cupron copper oxide containing solid surfaces possess comparable efficacy to the metallic hard surfaces, however represent a much more feasible alternative to the metallic surfaces as they are expected to be significantly more affordable and aesthetically more pleasing. Field trials are ongoing at the time of publication to demonstrate the efficacy of these countertops in a “real world” setting (Borkow and Monk, unpublished). The biocidal properties of copper oxide against a range of organisms have also been previously demonstrated [40-43].

Limitations of this study include that the data is based upon ATCC laboratory strains and conducted in a controlled setting such as a laboratory, however further work is ongoing utilizing field trials of the surface to demonstrate the efficacy in real world applications.

Bacterial resistance to biocidal control agents is of concern in infection prevention and can be exemplified by highly antibiotic resistant bacteria (with up to 2200-fold decreased sensitivity to the antibiotic (e.g. [44]) that have evolved in less than 50 years of antibiotic usage, making infected patient treatment extremely difficult (e.g. [45]). Consequently, the possibility of development of resistance to biocides is a real concern [46,47]. Importantly, as opposed to antibiotics, despite evolving in the continued presence of copper, no microorganisms that are highly resistant to copper have been found, but only microorganisms with increased copper tolerance [31]. Importantly, no resistant bacteria evolved *in vitro* when repeatedly and consecutively exposed to fabrics containing copper oxide particles [42]. The reason why no resistance to copper is found in microorganisms exposed to constant relatively high doses of copper, is because copper exerts its biocidal/antimicrobial activity not through one mechanism (as most antibiotics), but through several parallel non-specific mechanisms [48,49]. Both metallic copper and copper oxide particles, in the presence of humidity, even that present in air, release copper ions. The released copper ions can migrate and reach the microorganisms even though they may not be in direct contact with the copper oxide particles. These ions can cause plasma membrane permeabilization, membrane lipid peroxidation, alteration of proteins and inhibition of their biological assembly and activity, and denaturation of nucleic acids [48,49]. It is likely that the first site that copper ions damage is the microorganisms’ envelope via electrostatic forces [50], altering the membrane integrity and permeability [51,52]. Copper ions can also cause conformational changes in the structure of intracellular or membrane proteins or in the proteins active site also by direct interaction or by displacing essential metals from their native binding sites in the proteins (e.g. [53,54]). Furthermore, the redox cycling between Cu^2+^ and Cu^1+^, which can catalyze the production of highly reactive hydroxyl radicals, can subsequently damage lipids, proteins, DNA and other biomolecules [48,55]. In addition, metallic copper, as well as cuprous oxide particles, in the absence of humidity, cause massive membrane damage and kill microorganisms within minutes via direct “contact killing” [56-58]. Apparently, the metal-bacterial contact damages the cell envelope, which, in turn, makes the cells susceptible to further damage by copper ions [58].

Microorganisms cannot cope when exposed to high concentrations of copper and are irreversibly damaged, as demonstrated also in this study. Thus, the development of resistant bacteria to copper due to the introduction of the copper containing countertops to the hospital environment is not a concern.

With the ongoing HAI problem and the role of fomites and the environment being more clearly defined, the role of antimicrobial products with EPA approved public health claims, above and beyond the treated article claims and with clinical data supporting their role in HAI prevention, will become more important.

## Conclusion

The tested Cupron Enhanced EOS Surfaces containing copper oxide kill above 99.9% of a wide range of bacteria within two hours of exposure and continue to do so even after repeated contamination and multiple wet and dry abrasion cycles, passing all the acceptance criteria required by the EPA. These biocidal surfaces thus may be an important adjunct to be used in hospital settings to reduce environmental bioburden and potentially nosocomial infections.

## Abbreviations

CFU: Colony forming units; EDS: Energy-dispersive X-ray spectroscopy; EPA: Environmental Protection Agency; FBS: Fetal bovine serum; GLP: Good Laboratory Practice; HAI: Hospital Acquired Infections; MRSA: Methicillin resistant Staphylococcus aureus; PBS: Phosphate buffered solution; SEM: Scanning Electron Microscopy; TSA: Tryptic Soy Agar.

## Competing interests

KT is an employee of EOS Surfaces. ABM, VK and GB are employees of Cupron Inc. This study was funded by Cupron Inc. and EOS Surfaces that developed the antimicrobial surfaces.

## Authors’ contributions

ABM and GB made substantial contributions to conception, design, analysis and interpretation of data of the study, and writing the manuscript; VK and KT were key in designing and developing the test materials studied, and revising the manuscript critically for important intellectual content. All authors read and approved the final manuscript.

## References

[B1] Valles J Ferrer R Bloodstream infection in the ICU Infect Dis Clin North Am 2009 23 557 569 10.1016/j.idc.2009.04.005 19665083

[B2] Klevens RM Edwards JR Richards CL Jr Horan TC Gaynes RP Pollock DA Cardo DM Estimating health care-associated infections and deaths in U.S. hospitals, 2002 Public Health Rep 2007 122 160 166 17357358 10.1177/003335490712200205 PMC1820440

[B3] European Centre for Disease Prevention and Control Annual epidemiological report on communicable diseases in Europe 2010 Stockholm 22114980

[B4] Kock R Becker K Cookson B Gemert-Pijnen JE Harbarth S Kluytmans J Mielke M Peters G Skov RL Struelens MJ Tacconelli E Navarro TA Witte W Methicillin-resistant Staphylococcus aureus (MRSA): burden of disease and control challenges in Europe Euro Surveill 2010 15 19688 20961515 10.2807/ese.15.41.19688-en

[B5] Ferguson JK Preventing healthcare-associated infection: risks, healthcare systems and behaviour Intern Med J 2009 39 574 581 10.1111/j.1445-5994.2009.02004.x 19769680 PMC7165553

[B6] Gouvernement du Québec Loi modifiant la Loi sur les services de santé et les services sociaux concernant la prestation sécuritaire de services de santé et de services sociaux 2009

[B7] Hughes AJ Ariffin N Huat TL Abdul MH Hashim S Sarijo J Hughes AJ Ariffin N Huat TL Abdul Molok H Hashim S Sarijo J Abd Latif NH Abu Hanifah Y Kamarulzaman A Prevalence of nosocomial infection and antibiotic use at a university medical center in Malaysia Infect Control Hosp Epidemiol 2005 26 100 104 10.1086/502494 15693416

[B8] Rosenthal VD Maki DG Salomao R Moreno CA Mehta Y Higuera F Cuellar LE Arikan OA Abouqal R Leblebicioglu H Device-associated nosocomial infections in 55 intensive care units of 8 developing countries Ann Intern Med 2006 145 582 591 10.7326/0003-4819-145-8-200610170-00007 17043340

[B9] Rosenthal VD Device-associated nosocomial infections in limited-resources countries: findings of the International Nosocomial Infection Control Consortium (INICC) Am J Infect Control 2008 36 S171 12 19084148 10.1016/j.ajic.2008.10.009

[B10] Hidron AI Edwards JR Patel J Horan TC Sievert DM Pollock DA Fridkin SK NHSN annual update: antimicrobial-resistant pathogens associated with healthcare-associated infections: annual summary of data reported to the National Healthcare Safety Network at the Centers for Disease Control and Prevention, 2006–2007 Infect Control Hosp Epidemiol 2008 29 996 1011 10.1086/591861 18947320

[B11] Vincent JL Rello J Marshall J Silva E Anzueto A Martin CD Moreno R Lipman J Gomersall C Sakr Y Reinhart K International study of the prevalence and outcomes of infection in intensive care units JAMA 2009 302 2323 2329 10.1001/jama.2009.1754 19952319

[B12] Roberts RR Scott RD Hota B Kampe LM Abbasi F Schabowski S Ahmad I Ciavarella GG Cordell R Solomon SL Hagtvedt R Weinstein RA Costs attributable to healthcare-acquired infection in hospitalized adults and a comparison of economic methods Med Care 2010 48 1026 1035 10.1097/MLR.0b013e3181ef60a2 20940650

[B13] Curtis LT Prevention of hospital-acquired infections: review of non-pharmacological interventions J Hosp Infect 2008 69 204 219 10.1016/j.jhin.2008.03.018 18513830 PMC7172535

[B14] Dancer SJ White LF Lamb J Girvan EK Robertson C Measuring the effect of enhanced cleaning in a UK hospital: a prospective cross-over study BMC Med 2009 7 28 10.1186/1741-7015-7-28 19505316 PMC2700808

[B15] Hamilton D Foster A Ballantyne L Kingsmore P Bedwell D Hall TJ Hickok SS Jeanes A Coen PG Gant VA Performance of ultramicrofibre cleaning technology with or without addition of a novel copper-based biocide J Hosp Infect 2010 74 62 71 10.1016/j.jhin.2009.08.006 19819583

[B16] Pratt RJ Pellowe CM Wilson JA Loveday HP Harper PJ Jones SR McDougall C Wilcox MH epic2: national evidence-based guidelines for preventing healthcare-associated infections in NHS hospitals in England J Hosp Infect 2007 65 Suppl 1 S1 S64 17307562 10.1016/S0195-6701(07)60002-4 PMC7134414

[B17] Wren MW Rollins MS Jeanes A Hall TJ Coen PG Gant VA Removing bacteria from hospital surfaces: a laboratory comparison of ultramicrofibre and standard cloths J Hosp Infect 2008 70 265 271 10.1016/j.jhin.2008.07.017 18801594

[B18] Bhalla A Pultz NJ Gries DM Ray AJ Eckstein EC Aron DC Donskey CJ Acquisition of nosocomial pathogens on hands after contact with environmental surfaces near hospitalized patients Infect Control Hosp Epidemiol 2004 25 164 167 10.1086/502369 14994944

[B19] Boyce JM Potter-Bynoe G Chenevert C King T Environmental contamination due to methicillin-resistant Staphylococcus aureus: possible infection control implications Infect Control Hosp Epidemiol 1997 18 622 627 10.2307/30141488 9309433

[B20] Eckstein BC Adams DA Eckstein EC Rao A Sethi AK Yadavalli GK Donskey CJ Reduction of Clostridium Difficile and vancomycin-resistant Enterococcus contamination of environmental surfaces after an intervention to improve cleaning methods BMC Infect Dis 2007 7 61 10.1186/1471-2334-7-61 17584935 PMC1906786

[B21] Goodman ER Platt R Bass R Onderdonk AB Yokoe DS Huang SS Impact of an environmental cleaning intervention on the presence of methicillin-resistant Staphylococcus aureus and vancomycin-resistant enterococci on surfaces in intensive care unit rooms Infect Control Hosp Epidemiol 2008 29 593 599 10.1086/588566 18624666 PMC2670228

[B22] Hayden MK Bonten MJ Blom DW Lyle EA van de Vijver DA Weinstein RA Reduction in acquisition of vancomycin-resistant enterococcus after enforcement of routine environmental cleaning measures Clin Infect Dis 2006 42 1552 1560 10.1086/503845 16652312

[B23] Hota B Contamination, disinfection, and cross-colonization: are hospital surfaces reservoirs for nosocomial infection? Clin Infect Dis 2004 39 1182 1189 10.1086/424667 15486843 PMC7107941

[B24] Lu PL Siu LK Chen TC Ma L Chiang WG Chen YH Lin SF Chen TP Methicillin-resistant Staphylococcus aureus and Acinetobacter baumannii on computer interface surfaces of hospital wards and association with clinical isolates BMC Infect Dis 2009 9 164 10.1186/1471-2334-9-164 19796381 PMC2765444

[B25] Mutters R Nonnenmacher C Susin C Albrecht U Kropatsch R Schumacher S Quantitative detection of Clostridium difficile in hospital environmental samples by real-time polymerase chain reaction J Hosp Infect 2009 71 43 48 10.1016/j.jhin.2008.10.021 19041162

[B26] Sabino R Sampaio P Carneiro C Rosado L Pais C Isolates from hospital environments are the most virulent of the Candida parapsilosis complex BMC Microbiol 2011 11 180 10.1186/1471-2180-11-180 21824396 PMC3166928

[B27] Weber DJ Rutala WA Miller MB Huslage K Sickbert-Bennett E Role of hospital surfaces in the transmission of emerging health care-associated pathogens: norovirus, Clostridium difficile, and Acinetobacter species Am J Infect Control 2010 38 S25 S33 10.1016/j.ajic.2010.04.196 20569853

[B28] Young JM Naqvi M Richards L Microbial contamination of hospital bed handsets Am J Infect Control 2005 33 170 174 10.1016/j.ajic.2004.11.005 15798672

[B29] Champagne VK Helfritch DJ A demonstration of the antimicrobial effectiveness of various copper surfaces J Biol Eng 2013 7 8 10.1186/1754-1611-7-8 23537176 PMC3621704

[B30] Kramer A Schwebke I Kampf G How long do nosocomial pathogens persist on inanimate surfaces? A systematic review BMC Infect Dis 2006 6 130 138 10.1186/1471-2334-6-130 16914034 PMC1564025

[B31] Borkow G Monk AB Fighting nosocomial infections with biocidal non-intrusive hard and soft surfaces World J Clin Infect Dis 2012 12 77 90

[B32] Gould SWJ Fielder MD Kelly AF Morgan M Kenny J Naughton DP The antimicrobial properties of copper surfaces against a range of important nosocomial pathogens Anal Microbiol 2009 59 151 156 10.1007/BF03175613

[B33] Rai S Hirsch BE Attaway HH Nadan R Fairey S Hardy J Miller G Armellino D Moran WR Sharpe P Estelle A Michel JH Michels HT Schmidt MG Evaluation of the antimicrobial properties of copper surfaces in an outpatient infectious disease practice Infect Control Hosp Epidemiol 2012 33 200 201 10.1086/663701 22227992

[B34] Casey AL Adams D Karpanen TJ Lambert PA Cookson BD Nightingale P Nightingale P Miruszenko L Shillam R Christian P Elliott TS Role of copper in reducing hospital environment contamination J Hosp Infect 2010 74 72 77 10.1016/j.jhin.2009.08.018 19931938

[B35] Karpanen TJ Casey AL Lambert PA Cookson BD Nightingale P Miruszenko L Elliott TS The antimicrobial efficacy of copper alloy furnishing in the clinical environment: a crossover study Infect Control Hosp Epidemiol 2012 33 3 9 10.1086/663644 22173515

[B36] Marais F Mehtar S Chalkley L Antimicrobial efficacy of copper touch surfaces in reducing environmental bioburden in a South African community healthcare facility J Hosp Infect 2010 74 80 82 10.1016/j.jhin.2009.07.010 19781811

[B37] Schmidt MG Attaway HH Sharpe PA John J Jr Sepkowitz KA Morgan A Fairey SE Singh S Steed LL Cantey JR Freeman KD Michels HT Salgado CD Sustained reduction of microbial burden on common hospital surfaces through introduction of copper J Clin Microbiol 2012 50 2217 2223 10.1128/JCM.01032-12 22553242 PMC3405627

[B38] Efstathiou PA The role of antimicrobial copper surfaces in reducing healthcare associated infections Eur Infect Dis 2011 5 125 128

[B39] Salgado CD Sepkowitz KA John JF Cantey JR Attaway HH Freeman KD Sharpe PA Michels HT Schmidt MG Copper surfaces reduce the rate of healthcare-acquired infections in the intensive care unit Infect Control Hosp Epidemiol 2013 34 479 486 10.1086/670207 23571364

[B40] Borkow G Gabbay J Putting copper into action: copper-impregnated products with potent biocidal activities FASEB J 2004 18 1728 1730 15345689 10.1096/fj.04-2029fje

[B41] Borkow G Sidwell RW Smee DF Barnard DL Morrey JD Lara-Villegas HH Shemer-Avni Y Gabbay J Neutralizing viruses in suspensions by copper oxide based filters Antimicrob Agents Chemother 2007 51 2605 2607 10.1128/AAC.00125-07 17470650 PMC1913272

[B42] Borkow G Okon-Levy N Gabbay J Copper oxide impregnated wound dressings: biocidal and safety studies Wounds 2010 22 310 316 25901580

[B43] Borkow G Using copper to fight microorganisms Curr Chem Biol 2012 6 93 103 10.2174/187231312801254723

[B44] Goto H Shimada K Ikemoto H Oguri T Antimicrobial susceptibility of pathogens isolated from more than 10,000 patients with infectious respiratory diseases: a 25-year longitudinal study J Infect Chemother 2009 15 347 360 10.1007/s10156-009-0719-3 20012724

[B45] Durai R Ng PC Hoque H Methicillin-resistant Staphylococcus aureus: an update AORN J 2010 91 599 606 10.1016/j.aorn.2009.11.065 20451002

[B46] Maillard JY Bacterial resistance to biocides in the healthcare environment: should it be of genuine concern? J Hosp Infect 2007 65 Suppl 2 60 72 17540245 10.1016/S0195-6701(07)60018-8

[B47] Maillard JY Antimicrobial biocides in the healthcare environment: efficacy, usage, policies, and perceived problems Ther Clin Risk Manag 2005 1 307 320 18360573 PMC1661639

[B48] Borkow G Gabbay J Copper as a biocidal tool Curr Med Chem 2005 12 2163 2175 10.2174/0929867054637617 16101497

[B49] Borkow G Gabbay J An ancient remedy returning to fight microbial, fungal and viral infections Curr Chem Biol 2009 3 272 278

[B50] Nan L Liu Y Lu M Yang K Study on antibacterial mechanism of copper-bearing austenitic antibacterial stainless steel by atomic force microscopy J Mater Sci Mater Med 2008 19 3057 3062 10.1007/s10856-008-3444-z 18392666

[B51] Ohsumi Y Kitamoto K Anraku Y Changes induced in the permeability barrier of the yeast plasma membrane by cupric ion J Bacteriol 1988 170 2676 2682 3286617 10.1128/jb.170.6.2676-2682.1988 PMC211187

[B52] Avery SV Howlett NG Radice S Copper toxicity towards Saccharomyces cerevisiae: dependence on plasma membrane fatty acid composition Appl Environ Microbiol 1996 62 3960 3966 8899983 10.1128/aem.62.11.3960-3966.1996 PMC168214

[B53] Karlstrom AR Levine RL Copper inhibits the protease from human immunodeficiency virus 1 by both cysteine-dependent and cysteine-independent mechanisms Proc Natl Acad Sci U S A 1991 88 5552 5556 10.1073/pnas.88.13.5552 2062837 PMC51915

[B54] Karlstrom AR Shames BD Levine RL Reactivity of cysteine residues in the protease from human immunodeficiency virus: identification of a surface-exposed region which affects enzyme function Arch Biochem Biophys 1993 304 163 169 10.1006/abbi.1993.1334 8323281

[B55] Valko M Morris H Cronin MT Metals, toxicity and oxidative stress Curr Med Chem 2005 12 1161 1208 10.2174/0929867053764635 15892631

[B56] Espirito SC Lam EW Elowsky CG Quaranta D Domaille DW Chang CJ Bacterial killing by dry metallic copper surfaces Appl Environ Microbiol 2011 77 794 802 10.1128/AEM.01599-10 21148701 PMC3028699

[B57] Hans M Erbe A Mathews S Chen Y Solioz M Mucklich F Role of copper oxides in contact killing of bacteria Langmuir 2013 29 16160 16166 10.1021/la404091z 24344971

[B58] Mathews S Hans M Mucklich F Solioz M Contact killing of bacteria on copper is suppressed if bacterial-metal contact is prevented and is induced on iron by copper ions Appl Environ Microbiol 2013 79 2605 2611 10.1128/AEM.03608-12 23396344 PMC3623184

